# Supplementary material for: Deletion of wfs1 Impairs Oligodendrocyte Precursor Cells Dorsal Distribution and Myelination Through the wfs1-hmgcs1 Axis in Zebrafish
Source: Biology (Basel). 2026 Aug 16;15(16):1402. doi: 10.3390/biology15161402 (PMC13510151; doi:10.3390/biology15161402)
Supplement: Supplementary file 1 [file biology-15-01402-s001.zip › File S1-Transcriptome sequencing data and differentially expressed genes (DEGs) in wfs1-- zebrafish at 5 dpf/1.GO/wfs1KOvsWT/ALL/wfs1KOvsWT.all_GObar_graph_updown.pdf]

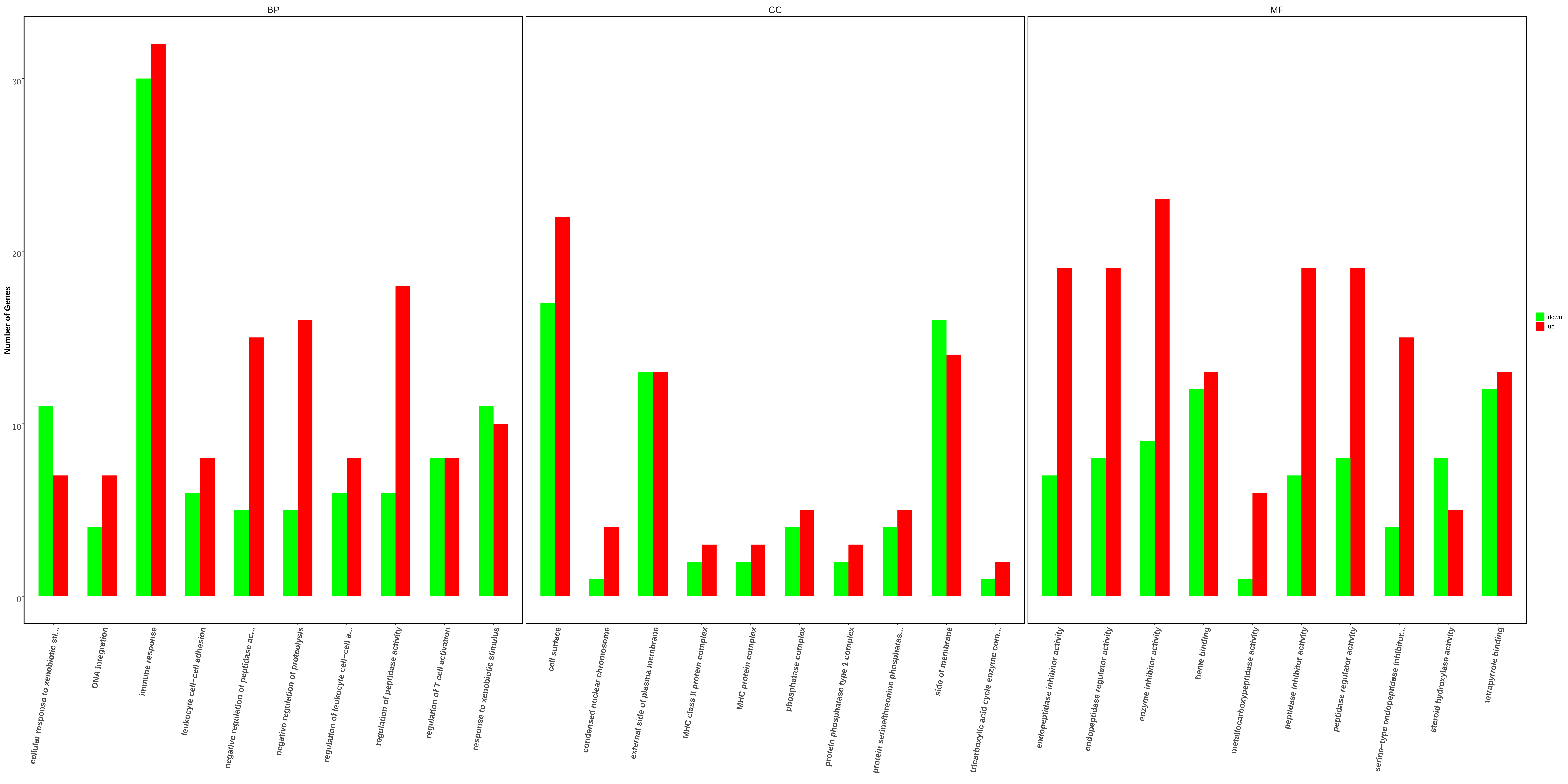

BP
CC
MF
Number of Genes
0
10
20
30
down
up
cellular response to xenobiotic sti...
DNA integration
immune response
leukocyte cell−cell adhesion
negative regulation of peptidase ac...
negative regulation of proteolysis
regulation of leukocyte cell−cell a...
regulation of peptidase activity
regulation of T cell activation
response to xenobiotic stimulus
cell surface
condensed nuclear chromosome
external side of plasma membrane
MHC class II protein complex
MHC protein complex
phosphatase complex
protein phosphatase type 1 complex
protein serine/threonine phosphatas...
side of membrane
tricarboxylic acid cycle enzyme com...
endopeptidase inhibitor activity
endopeptidase regulator activity
enzyme inhibitor activity
heme binding
metallocarboxypeptidase activity
peptidase inhibitor activity
peptidase regulator activity
serine−type endopeptidase inhibitor...
steroid hydroxylase activity
tetrapyrrole binding
